# The Need for Young Qualified Medical Men

**Published:** 1914-12-19

**Authors:** Donald MacAlister

**Affiliations:** President, G.M.C. Medical Council Office, 299 Oxford Street, London


### THE EDITOR'S LETTER-BOX.

### The Need for Young Qualified Medical Men.

*To the Editor of* THE HOSPITAL.

SIR,—In my Presidential Address to the General Medical Council on November 24 I expressed the opinion that "the need for efficient physicians and surgeons, in the field and at home, is not less urgent than the need for efficient soldiers and sailors"; and I said that I had "felt it my duty to press this consideration on senior students, who, though they have nearly completed their curriculum, are ready to forgo the prospect of early qualification and to enrol themselves straightway in the combatant forces."

To-day I have received a letter from Surgeon-General Sir Alfred Keogh, which contains the following passage: "I think with you that the senior student is best fulfilling his duty to the country by getting his degree, and then joining the Army. The need for young qualified men will become great, and I should regret that the supply should be diminished."

As I daily receive letters from senior students and their parents, who desire guidance in their choice of apparently conflicting duties, I shall be grateful if you will make known the opinion held at the War Office on the subject.—I am, yours faithfully,

DONALD MACALISTER, President, G.M.C.
Medical Council Office,
299 Oxford Street, London,
December 10, 1914.

